# Length of hospital stay after hip fracture and readmission rates of persons with and without Alzheimer’s disease: a matched cohort study

**DOI:** 10.1186/s12877-020-01609-5

**Published:** 2020-06-18

**Authors:** Blair Rajamaki, Marjaana Koponen, Sirpa Hartikainen, Anna-Maija Tolppanen

**Affiliations:** 1 School of Pharmacy, Faculty of Health Sciences, Kuopio Campus, University of Eastern Finland, P.O. Box 1627, FI-70211 Kuopio, Finland; 2 Kuopio Research Centre of Geriatric Care, University of Eastern Finland, Kuopio, Finland; 3 Centre for Medicine Use and Safety, Faculty of Pharmacy and Pharmaceutical Sciences, Monash University, Parkville, VIC Australia

**Keywords:** Alzheimer’s disease, Length of stay, Hip fracture, Readmission

## Abstract

**Background:**

Hospital length of stays (LOS) for incident of hip fracture are decreasing, but it is unknown if these changes have negative impacts on vulnerable older patient populations, like those with Alzheimer’s disease (AD). We aimed to assess if persons with and without AD have different hospital LOS for hip fracture, and is the LOS associated with hospital readmissions.

**Methods:**

Utilizing register-based data for a matched cohort study nested in the Medication use and Alzheimer’s disease study (MEDALZ), we collected all community-dwelling persons in Finland diagnosed with AD during 2005–2012, had incident of first hip fracture between 2005 and 2015 after AD diagnosis, and were discharged alive from an acute care hospital. Hospital LOS and hospital readmissions within 30-days and 90-days were compared between those with and without AD and risk of readmission was assessed using binary logistic regression analysis.

**Results:**

In this matched cohort study of 12,532 persons (mean age 84.6 years (95% CI: 84.5–84.7), 76.8% women), the median LOS in an acute care hospital was 1 day shorter for those with AD (median 4 days, IQR 3–7) than those without AD (median 5 days, IQR 3–7) (*P* < 0.001). However, the AD cohort had respectively 6 days and 5 days longer median LOS in a community hospital, and total hospital stay compared to the non-AD cohort (*P* < 0.001 for all comparisons). Those with AD had fewer readmissions within 30-days (10.7%) and 90-days (16.9%) compared to those without AD (13.3% 30-days and 20.7% 90-days) (*P* < 0.001 for all comparisons). Both cohorts had a reduced readmission risk within 30-days when the LOS in an acute care hospital was 4–14 days, compared to a LOS less than 4 days.

**Conclusions:**

Persons with AD had shorter acute care hospital LOS, but had longer LOS in a community hospital setting compared to those without AD, which is similar to other findings when comparing total hospital LOS. These findings imply that short LOS in acute care hospitals may be associated with poor health outcomes for vulnerable older populations after hip fracture.

## Background

Alzheimer’s disease (AD) is a common neurodegenerative disease, usually affecting the older adult population and is the most prevalent form of dementia. The progressive and chronic course of this incurable disease is burdening healthcare systems globally [1]. Persons with AD are at a higher risk of falls compared to persons without dementia, [2] often leading to hip fractures [3]. Outcomes after hip fracture are often negative for the individual, including loss of function and mobility, and increased mortality, but also are burdensome for the health care system due to high care costs. As the population ages throughout the world, incidence of both hip fracture and AD are also on the rise. Scandinavia, Finland included, has the highest reported incidence of hip fractures in the world [4].

Hip fractures have been used as a “tracer condition” to monitor healthcare response when designing clinical and organizational improvements in the quality and effectiveness of care for older populations [5]. Quality indicators for hip fracture care, such as length of stay (LOS) and hospital readmissions, are measurable aspects that reflect the quality of care and are commonly used outcome measures [6].

Definitions of LOS are varied. Several studies have reported on the acute surgical phase of treatment, while others have included rehabilitation or in-patient care. A systematic review of observational studies found longer hospital LOS after hip fracture for persons with dementia [7], while other studies found shorter LOS compared to those without dementia [8, 9]. Comparing hospital LOS in studies is difficult due different care models utilized in health care systems.

Hospital readmissions have been increasingly accepted as a metric for quality of care because they may be seen as a preventable failure to ensure safe discharge [10, 11]. A systematic review found the effect of cognitive disorders on hospital readmissions after hip fracture to be conflicting; some observed increased risk of readmission, while others a decrease in risk [12]. Patients readmitted within 30-days of discharge after a hip fracture have been observed to have worse outcomes, with nearly two times higher mortality rate compared to those without readmissions during the first year [13, 14].

Further research is needed on the relationship of AD, hip fractures, and the LOS during the acute surgical phase of treatment and inpatient treatment, along with the 30-day and 90-day hospital readmissions to properly support this vulnerable population. The main aim of this study is to compare the LOS of those with and without AD for the acute care hospital stay, inpatient treatment in a community hospital, and the total hospital LOS using the data collected in the Finnish nationwide Medication use and Alzheimer’s disease (MEDALZ) study of persons with AD. The second aim of the study is to compare the 30-day and 90-day readmission rates of persons with and without AD after incident of hip fracture and the possible association of LOS and readmission rates of those with and without AD.

## Methods

### Study population

This retrospective matched cohort study was nested in the MEDALZ study which included all community-dwelling persons who received a new clinically verified diagnosis of AD from 2005 to 2011 (*N* = 70,719) in Finland. Age in the MEDALZ cohort ranges from 34 to 105 years (mean 80.1 (standard deviation 7.1) years) and 65.2% were women. Those with an AD diagnosis were identified from the Finnish Special Reimbursement Register (FSRR), which is maintained by the Social Insurance Institution of Finland (SII) as described in a previous article [15]. Persons diagnosed with certain chronic diseases, such as AD, are eligible for higher reimbursement of their medications and this data is recorded by the FSRR.

For a person to be eligible for the FSRR for AD they need a verified diagnosis of AD written in a medical statement by their physician and submitted to SII. The medical statement must confirm that the patient has: 1) symptoms consistent with AD, 2) experienced a decrease in social capacity over a period of at least 3 months, 3) received a computed tomography (CT)/ magnetic resonance imaging scan (MRI) to confirm that neuroanatomical changes are consistent with AD, 4) had possible alternative diagnoses excluded, and 5) received confirmation of the diagnosis by a registered neurologist or geriatrician. Along with the medical statement submitted by the physicians to SII, findings from the CT/MRI, laboratory tests, cognitive tests and statements from the patient and their family are included. The AD diagnosis was based mainly on the National Institute of Neurological and Communicative Disorders and Stroke and the Alzheimer’s Disease and Related Disorders Association’s (NINCDS-ADRDA) and Diagnostic and Statistical Manual of Mental Disorders, 4th edition (DSM-IV) criteria for Alzheimer’s disease [16, 17].

Identification of hip fractures between 1972 and 2015 was gathered from the National Hospital Discharge Register using the International Classification of Diseases (ICD) -10 codes: S72.0 (fracture of neck of femur), S72.1 (pertrochanteric fracture) and S72.2 (subtrochanteric fracture). The corresponding ICD-9 and ICD-8 codes were used to exclude previous hip fractures. Information on inpatient and outpatient use of healthcare services of individuals is collected by the Finnish Care Register for Health Care and the data are continuously updated. When comparing audit and register-based data, 98.1% of occurred hip fractures were recorded in the inpatient data of the Care Register for Health Care [18]. Only those that had sustained their first hip fracture after their verified AD diagnosis and were discharged alive from the hospital were included in the study. The Causes of Death Register, maintained by Statistics Finland, reported the data on mortality from 2005 to 2015. Similar methods have been published previously [19].

To compare the hospital LOSs and readmission rates among persons with and without AD, an age, sex, and university hospital district-matched cohort of persons who did not have a clinically verified AD diagnosis, but had sustained their first hip fracture between 2005 and 2015, was identified from a SII database. The SII database covers all residents of Finland who are eligible for social security.

Data from the various national registers was compiled using a unique personal identity code assigned to every resident of Finland and has previously been described [20]. De-identification (i.e., substitution of anonymous numerical codes for the personal identity codes) of the all the data were completed by the register maintainers before being released to the research team. Study participants were not contacted. Ethics committee approval or informed consent were not required according to the Finnish legislation. The MEDALZ study protocol was approved by the register maintainers (Statistics Finland, SII, and National Institute of Health and Welfare) and the University of Eastern Finland.

**Fig. 1:**
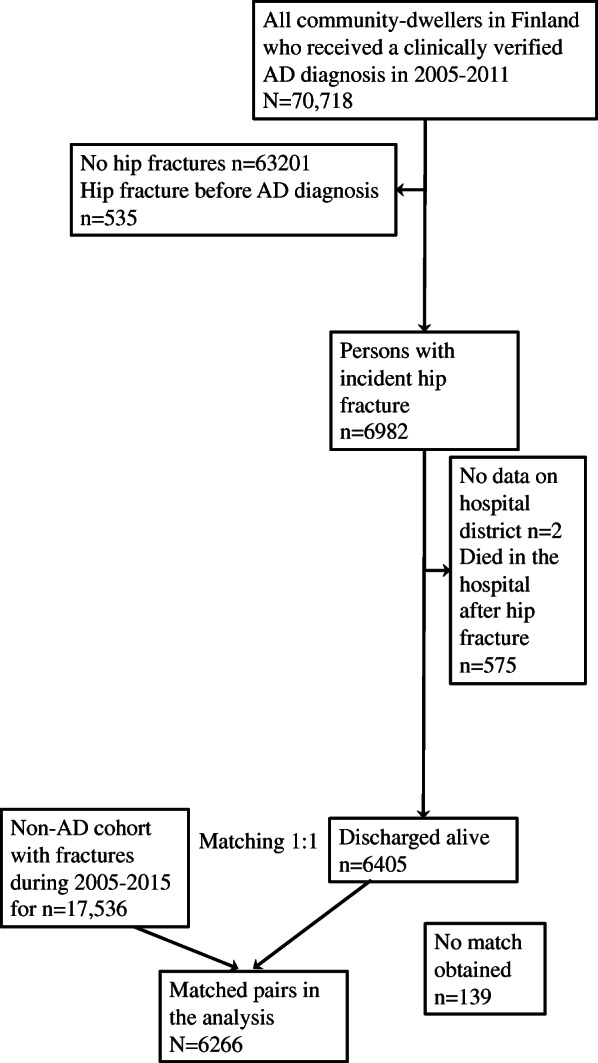
Flow chart of the study population exclusions and matched comparisons

Of those diagnosed with AD from 2005 to 2011, 6982 persons sustained their first hip fracture from 2005 to 2015 after their AD diagnosis. Two persons were excluded due to no data available on their hospital district and 575 persons died during the initial hospital period. No matches were obtained for 139 persons with a hip fracture and verified AD diagnosis. A total of 6266 matched pairs were used for the analysis **(**Fig. 1**).**

### Length of acute care and community hospital stay

The LOS (in days) in an acute care setting was collected from The Care Register for Health Care and was calculated from the day of admission to the day of discharge and considered the acute care hospital stay. Community hospitals are often utilized for patients after being discharged from an acute care hospital for rehabilitation services post fracture and care of contingent conditions. The LOS in a community hospital was interrupted in some cases by a need for hospital readmission but could have continued when discharged from readmission stay at the acute care hospital. The community hospital stay days before and after readmission were included in calculating community hospital LOS (Additional File 1: Figure S1). The total LOS was calculated from adding the acute care hospital LOS for hip fracture, and the cumulative community hospital days.

Acute care hospital readmissions were also identified from The Care Register for Health Care within 30-days and within 90-days of discharge from the initial acute care hospital stay.

### Covariates

Comorbidity history (since 1972) prior to the incident of hip fracture were identified from the National Hospital Discharge Register and Special Reimbursement Register. Diabetes, cardiovascular diseases (CVD) including, hypertension, coronary artery disease, familial hypercholesterolemia, heart failure, and chronic cardiac arrhythmias, epilepsy, and asthma or chronic obstructive pulmonary disease (COPD) data were obtained from the Special Reimbursement Register. Also obtained from the National Hospital Discharge Register was data on stroke (ICD-10 codes I60-I64), mental disorders (ICD-10 codes F04-F99), and required level of assistance at discharge.

We used occupational social class to represent the socioeconomic position (SEP) which was obtained from the censuses maintained by Statistics Finland. Data was collected every 5 years from 1970 to 1990, followed by collections in 1993, 1995, 2000, and then annually from 2004 onwards. The 2010 version of the original classification is available from reference [21]. The occupational social class categories were created and included “managerial/professional”, “office worker”, “farming/forestry”, “sales/industry/cleaning”, “unknown” and “did not respond”. Individuals were categorized to their work type during their middle age (45–55 years old) because people retired at lower ages previously.

### Statistical analyses

Characteristics between AD and non-AD cohorts were compared with Fischer’s Exact test (2-sided) or Χ^2^ test for categorical variables. Differences in LOS were compared with Mann-Whitney U test for statistical significance. Analyses were performed separately for the AD and non-AD cohorts. To compare the AD and non-AD cohorts’ LOS (acute care hospital stay, community hospital stay, and total hospital stay) and 30-day and 90-day hospital readmissions, binary logistic regression analysis was used with adjusted models. All statistical analyses were completed using SPSS version 24.0 for Mac (IBM Corp., Armonk, NY).

## Results

### Study population

The mean age of the study sample (76.8% women) was 84.6 years (SD: 5.7) **(**Table 1). Between the AD and non-AD hip fracture cohorts there were no regional differences, furthermore no differences were observed between the month (data not shown) or year when the hip fracture occurred. A history of a mental disorder was more prevalent in the AD cohort, while stroke and cardiovascular disease were more prevalent in the non-AD cohort. The prevalence of asthma/COPD, diabetes, and epilepsy were similar in both cohorts. There were slight differences in the distribution of occupational social class between cohorts with no consistent pattern. Altogether 3.9% of those with AD and 3.2% of those without AD died within 30 days after discharge from their acute care hospital stay, and a larger difference was observed with 90-day mortality of 11.3% and 7.6%, respectively.

**Table 1:** Characteristics of AD and non-AD cohorts with incident hip fracture

Characteristics		AD Cohort (*n* = 6266)	Non-AD Cohort (*n* = 6266)	***P***-Value
Age at hip fracture, mean ± SD		84.6 ± 5.7	84.6 ± 5.7	Matched 1.00
Sex				Matched 1.00
	Female, n (%)	4815 (76.8%)	4815 (76.8%)	··
	Male, n (%)	1451 (23.2%)	1451 (23.2%)	··
University Hospital District				0.29
	Helsinki, n (%)	1817 (29.0%)	1791 (28.6%)	··
	Kuopio, n (%)	1230 (19.6%)	1195 (19.1%)	··
	Oulu, n (%)	882 (14.1%)	839 (13.4%)	··
	Tampere, n (%)	1354 (21.6%)	1381 (22.0%)	··
	Turku, n (%)	983 (15.7%)	1060 (16.9%)	··
Year of hip fracture				0.99
	2005, n (%)	67 (1.1%)	73 (1.2%)	··
	2006, n (%)	242 (3.9%)	235 (3.8%)	··
	2007, n (%)	357 (5.7%)	358 (5.7%)	··
	2008, n (%)	562 (9.0%)	563 (9.0%)	··
	2009, n (%)	603 (9.6%)	606 (9.7%)	··
	2010, n (%)	769 (12.3%)	787 (12.6%)	··
	2011, n (%)	895 (14.3%)	869 (13.9%)	··
	2012, n (%)	904 (14.4%)	894 (14.3%)	··
	2013, n (%)	788 (12.6%)	783 (12.5%)	··
	2014, n (%)	592 (9.4%)	595 (9.5%)	··
	2015, n (%)	487 (7.8%)	503 (8.0%)	··
Median acute care hospital stay, days (IQR)		4 (3–7)	5 (3–7)	0.001
Discharged to community hospital, n (%)		5486 (87.6%)	5483 (87.5%)	0.93
Median community hospital stay, days (IQR) ^a^		35(16–84)	29 (15–65)	< 0.001
Median total hospital stay, median (IQR)		34 (15–81)	29 (15–63)	< 0.001
Readmission within 30-days, n (%)		672 (10.7%)	836 (13.3%)	< 0.001
Readmission within 90-days, n (%)		1059 (16.9%)	1300 (20.7%)	< 0.001
Died within 30-days, n (%)		246 (3.9%)	200 (3.2%)	0.03
Died within 90-days, n (%)		705 (11.3%)	478 (7.6%)	< 0.001
Diabetes, n (%)		737 (11.8%)	774 (12.4%)	0.32
Stroke, n (%)		546 (8.7%)	646 (10.3%)	0.003
CVD, n (%)		3018 (48.2%)	3152 (50.3%)	0.017
Mental disorders, n (%)		1464 (23.4%)	801 (12.8%)	< 0.001
Asthma/COPD, n (%)		539 (8.6%)	573 (9.1%)	0.28
Epilepsy, n (%)		165 (2.6%)	135 (2.2%)	0.09
Highest occupational social class				0.005
	Managerial/ Professional, n (%)	1170 (18.7%)	1135 (18.1%)	··
	Office worker, n (%)	608 (9.7%)	595 (9.5%)	··
	Farming/ forestry, n (%)	1113 (17.8%)	1258 (20.1%)	··
	Sales/industry/ cleaning, n (%)	2648 (42.3%)	2503 (39.9%)	··
	Unknown, n (%)	671 (10.7%)	701 (11.2%)	··
	Did not respond, n (%)	56 (0.9%)	74 (1.2%)	··
Median duration of AD diagnosis, years (IQR)		2.8 (1.3–4.4)	NA	NA
Required level of assistance at discharge				< 0.001
	Nearly independent, n (%)	143 (2.3%)	325 (5.2%)	··
	Intermittent need, n (%)	802 (12.8%)	1142 (18.2%)	··
	Recurrent need, n (%)	2708 (43.2%)	2795 (44.6%)	··
	Nearly continuous, n (%)	1387 (22.1%)	1033 (16.5%)	··
	Continuous, n (%)	946 (15.1%)	708 (11.3%)	··
	Missing data, n (%)	280 (4.5%)	263 (4.2%)	··

*AD* Alzheimer’s Disease; *SD* Standard Deviation; *IQR* Interquartile Range; *NA* Not Applicable; *CVD* Cardiovascular Disease; *COPD* Chronic Obstructive Pulmonary Disease

^a^only those discharged to a community hospital

### Length of stay

The median LOS for the acute care hospital stay for the AD cohort was 4 days compared to non-AD cohort’s LOS of 5 days. Upon discharge from the acute care hospital stay, about 87% of each cohort discharged to a community hospital setting for further care (Table 1). The required level of assistance at discharge varied between the two cohorts with a greater proportion of the AD cohort needing continuous or nearly continuous assistance compared to the non-AD cohort (Table 1). The AD population median LOS in those discharged to a community hospital was 6 days longer compared to the non-AD persons. The total hospital LOS (combined acute care and community hospital LOS) for those with AD being approximately 5 days longer than those without AD.

### 30-day and 90-day hospital readmission

Hospital readmissions within 30-days of acute care hospital stay discharge were less common in the AD cohort (10.7% re-hospitalized) in comparison to the non-AD cohort (13.3% re-hospitalized) (Table 2). Differences between the AD and non-AD cohorts remained after adjustment for sex, age, hospital district, year of hip fracture, occupational social class, comorbidities, and required level of assistance at discharge. Similar results were observed within 90-days hospital readmissions (16.9% of the AD cohort and 20.7% of the non-AD cohort) with significant differences between cohorts remaining after adjustment of all covariates. In both cohorts a history of diabetes, cardiovascular disease, and epilepsy were more prevalent in those who were readmitted within 30-days and within 90-days compared to those not readmitted (Additional File 1: Tables S1-S4). Those who were readmitted within 30-days had a higher mortality within 90-days and higher required level of assistance at discharge. A history of stroke was associated with higher 90-day readmission risk in persons with AD.

**Table 2:** Relative risk of 30-day and 90-day readmission in AD and non-AD cohorts after the acute care hospital stay

	n of Readmissions / n (%)	RR (95% CI) Unadjusted	RR (95% CI)^a^	RR (95% CI) ^b^
**30-day Readmission**				
Non-AD Cohort (Ref.)	836 /6266 (13.3)	1.00	1.00	1.00
AD Cohort	672 /6266 (10.7)	0.78 (0.70–0.87)	0.78 (0.70–0.87)	0.76 (0.68–0.85)
**90-day Readmission**				
Non-AD Cohort (Ref.)	1300/6266 (20.7)	1.00	1.00	1.00
AD Cohort	1059/6266 (16.9)	0.78 (0.71–0.85)	0.77 (0.71–0.85)	0.77 (0.70–0.84)

Abbreviations: *AD* Alzheimer’s disease; *RR* risk ratio; *CI* Confidence Interval; *Ref.* Reference

^a^ adjusted for age, sex, hospital district, and year of hip fracture

^b^ adjusted for age, sex, hospital district, diabetes, stroke, cardiovascular disease, mental disorders, highest occupational social class, duration of AD on hip fracture (in years), and required level of assistance at discharge.

### Comparing hospital readmissions to length of stay

In both the AD and non-AD cohort, persons with an acute care hospital LOS of 4–7 days or 8–14 days were less likely to have been readmitted within 30-days than those who had an acute care hospital stay less than 4 days (Table 3). A lower risk of readmission within 90-days continued to be observed with 4–14-day acute care stays in the AD cohort, but not in the non-AD cohort. However, the trend for lower risk of 90-day readmission with longer acute care hospital stays was also observed among those without AD, but the confidence intervals overlapped.

**Table 3:** Duration of Acute care hospital stay compared to risk of 30-day and 90-day hospital readmission in AD and non-AD cohorts

			< 4 days (Ref.)	4–7 days	8–14 days	≥ 15 days
**30-day Readmission**						
AD Cohort	n Readmitted/ n (%)	672/6266 (10.7)	399/3233 (12.3)	153/1814 (8.4)	79/877 (9.0)	41/342 (12.0)
	RR (95% CI) Unadjusted		1.00	0.65 (0.54–0.80)	0.70 (0.55–0.91)	0.97 (0.69–1.36)
	RR (95% CI) ^a^		1.00	0.68 (0.55–0.83)	0.72 (0.55–0.93)	0.95 (0.67–1.34)
	RR (95% CI) ^b^		1.00	0.67 (0.54–0.82)	0.72 (0.55–0.94)	0.89 (0.63–1.30)
Non-AD Cohort	n Readmitted/ n (%)	836/6266 (13.3)	463/3023 (15.3)	233/1955 (11.9)	103/968 (10.6)	37/320 (11.6)
	RR (95% CI) Unadjusted		1.00	0.75 (0.63–0.89)	0.66 (0.52–0.83)	0.72 (0.51–1.03)
	RR (95% CI) ^a^		1.00	0.79 (0.67–0.94)	0.69 (0.55–0.87)	0.73 (0.51–1.05)
	RR (95% CI) ^b^		1.00	0.82 (0.69–0.98)	0.72 (0.57–0.91)	0.72 (0.50–1.04)
**90-day Readmission**						
AD Cohort	n Readmitted/ n (%)	1059/6266 (16.9)	586/3233 (18.1)	276/1814 (15.2)	128/877 (14.6)	69/342 (20.0)
	RR (95% CI) Unadjusted		1.00	0.81 (0.69–0.95)	0.77 (0.63–0.95)	1.14 (0.86–1.51)
	RR (95% CI) ^a^		1.00	0.83 (0.71–0.98)	0.79 (0.63–0.98)	1.12 (0.85–1.49)
	RR (95% CI) ^b^		1.00	0.83 (0.70–0.97)	0.80 (0.64–0.99)	1.11 (0.84–1.49)
Non-AD Cohort	n Readmitted/ n (%)	1300/6266 (20.7)	655/3023 (21.7)	403/1955 (20.6)	181/968 (18.7)	61/320 (19.1)
	RR (95% CI) Unadjusted		1.00	0.94 (0.82–1.08)	0.83 (0.70–0.99)	0.85 (0.64–1.14)
	RR (95% CI) ^a^		1.00	0.97 (0.84–1.12)	0.85 (0.71–1.03)	0.85 (0.63–1.14)
	RR (95% CI) ^b^		1.00	0.99 (0.86–1.14)	0.88 (0.73–1.07)	0.87 (0.64–1.17)

Abbreviations: *AD* Alzheimer’s disease; *RR* risk ratio; *CI* Confidence Interval; *Ref.* Reference

^a^ adjusted for age, sex, hospital district, and year of hip fracture

^b^ adjusted for age, sex, hospital district, year of hip fracture, diabetes, stroke, cardiovascular disease, mental disorders, highest occupational social class, duration of AD on hip fracture (in years), and required level of assistance at discharge

LOS in a community hospital was not associated with readmission within 30-days and 90-days in the AD cohort (Table 4). However, in those without AD, those with LOS greater than 7 days compared to a shorter LOS had a decreased risk of 30-day readmission. A decrease in risk of readmission within 90-days for the non-AD cohort continued to be observed for a LOS of 8–14 days and 15–29 days in the non-AD cohort.

**Table 4:** Duration of Community hospital stay compared to risk of 30-day and 90- day hospital readmission in AD and non-AD cohorts

			< 8 days (Ref.)	8–14 days	15–29 days	≥ 30 days
**30-day Readmission**						
AD Cohort	n Readmitted/ n (%)^a^	606/5486 (11.0)	64/533 (12.0)	85/725 (11.7)	109/1180 (9.2)	348/3048 (11.4)
	RR (95% CI) Unadjusted		1.00	0.97 (0.69–1.38)	0.75 (0.54–1.03)	0.95 (0.71–1.26)
	RR (95% CI) ^b^		1.00	0.97 (0.69–1.38)	0.75 (0.54–1.04)	0.93 (0.70–1.24)
	RR (95% CI) ^c^		1.00	0.99 (0.70–1.40)	0.76 (0.54–1.05)	0.92 (0.69–1.24)
Non-AD Cohort	n Readmitted/ n (%)^c^	753/5483 (13.7)	99/515 (19.2)	99/847 (11.7)	154/1384 (11.1)	401/2737 (14.7)
	RR (95% CI) Unadjusted		1.00	0.56 (0.41–0.75)	0.53 (0.40–0.69)	0.72 (0.57–0.92)
	RR (95% CI) ^b^		1.00	0.58 (0.42–0.78)	0.56 (0.42–0.74)	0.77 (0.60–0.98)
	RR (95% CI) ^c^		1.00	0.57 (0.42–0.77)	0.55 (0.42–0.73)	0.74 (0.58–0.95)
**90-day Readmission**						
AD Cohort	n Readmitted/ n (%)^c^	958/5486 (17.5)	93/533 (17.4)	117/725 (16.1)	186/1180 (15.8)	562/3048 (18.4)
	RR (95% CI) Unadjusted		1.00	0.91 (0.68–1.23)	0.89 (0.67–1.23)	1.07 (0.84–1.36)
	RR (95% CI) ^b^		1.00	0.91 (0.67–1.23)	0.89 (0.67–1.17)	1.06 (0.83–1.36)
	RR (95% CI) ^c^		1.00	0.92 (0.68–1.24)	0.90 (0.68–1.18)	1.06 (0.83–1.36)
Non-AD Cohort	n Readmitted/ n (%)^c^	1179/5483 (21.5)	125/515 (24.3)	158/847 (18.7)	253/1384 (18.3)	643/2737 (23.5)
	RR (95% CI) Unadjusted		1.00	0.72 (0.55–0.93)	0.70 (0.55–0.89)	0.96 (0.77–1.19)
	RR (95% CI) ^b^		1.00	0.72 (0.55–0.95)	0.72 (0.56–0.92)	1.00 (0.80–1.24)
	RR (95% CI) ^c^		1.00	0.71 (0.55–0.93)	0.71 (0.56–0.92)	0.97 (0.78–1.22)

Abbreviations: *AD* Alzheimer’s disease; *RR* risk ratio; *CI* Confidence Interval; *Ref.* Reference

^a^ Only those discharged to a community hospital after initial acute care hospital stay

^b^ adjusted for age, sex, hospital district, and year of hip fracture.

^c^ adjusted for age, sex, hospital district, year of hip fracture, diabetes, stroke, cardiovascular disease, mental disorders, highest occupational social class, duration of AD on hip fracture (in years), and required level of assistance at discharge.

No differences in risk of readmission within 30-days and 90-days were observed in the AD cohort for any categorized total hospital LOS (Table 5). A decrease in risk of readmission within 30-days was observed in the non-AD cohort when the total hospital LOS was 10–19 days compared to a LOS less than 10 days, but no difference in risk was observed with a longer LOS. No difference in risk of readmission within 90-days was observed in the non-AD cohort in the adjusted model.

**Table 5:** Duration of Total hospital stay compared to risk of 30-day and 90-day hospital readmission in AD and non-AD cohorts

			< 10 days (Ref.)	10–19 days	20–29 days	≥ 30 days
**30-day Readmission**						
AD Cohort	n Readmitted/ n (%)	672/6266 (10.7)	110/1047 (10.5)	102/1002 (10.2)	85/812 (10.5)	375/3405 (11.0)
	RR (95% CI) Unadjusted		1.00	0.97 (0.73–1.28)	0.99 (0.74–1.28)	1.05 (0.84–1.32)
	RR (95% CI) ^a^		1.00	0.90 (0.68–1.21)	0.93 (0.69–1.26)	0.96 (0.76–1.21)
	RR (95% CI) ^b^		1.00	0.87 (0.65–1.16)	0.90 (0.66–1.22)	0.90 (0.71–1.14)
Non-AD Cohort	n Readmitted/ n (%)	836/6266 (13.3)	145/996 (14.6)	130/1114 (11.7)	132/1027 (12.9)	429/3129 (13.7)
	RR (95% CI) Unadjusted		1.00	0.78 (0.60–0.99)	0.87 (0.67–1.11)	0.93 (0.76–1.14)
	RR (95% CI) ^a^		1.00	0.75 (0.58–0.97)	0.88 (0.68–1.13)	0.94 (0.76–1.15)
	RR (95% CI) ^b^		1.00	0.73 (0.56–0.94)	0.85 (0.65–1.10)	0.88 (0.71–1.09)
**90-day Readmission**						
AD Cohort	n Readmitted/ n (%)	1059/6266 (16.9)	158/1047 (15.1)	153/1002 (15.3)	132/812 (16.3)	616/3405 (18.1)
	RR (95% CI) Unadjusted		1.00	1.01 (0.80–1.29)	1.09 (0.85–1.41)	1.24 (1.03–1.50)
	RR (95% CI) ^a^		1.00	0.97 (0.76–1.24)	1.05 (0.81–1.35)	1.17 (0.96–1.42)
	RR (95% CI) ^b^		1.00	0.94 (0.74–1.20)	1.02 (0.79–1.32)	1.12 (0.92–1.37)
Non-AD Cohort	n Readmitted/ n (%)	1300/6266 (20.7)	194/996 (19.5)	196/1114 (17.6)	200/1027 (19.5)	710/3129 (22.7)
	RR (95% CI) Unadjusted		1.00	0.88 (0.71–1.10)	1.00 (0.80–1.25)	1.21 (1.02–1.45)
	RR (95% CI) ^a^		1.00	0.86 (0.69–1.07)	1.00 (0.80–1.25)	1.22 (1.02–1.47)
	RR (95% CI) ^b^		1.00	0.84 (0.67–1.05)	0.97 (0.77–1.21)	1.16 (0.97–1.40)

Abbreviations: *AD* Alzheimer’s disease; *RR* risk ratio; *CI* Confidence Interval; *Ref.* Reference

^a^ adjusted for age, sex, hospital district, and year of hip fracture

^b^ adjusted for age, sex, hospital district, year of hip fracture, diabetes, stroke, cardiovascular disease, mental disorders, highest occupational social class, duration of AD on hip fracture (in years), and required level of assistance at discharge.

## Discussion

This large, population-based cohort study found persons with AD had a 1 day shorter LOS in an acute care hospital, but the total hospital LOS was 5 days longer (when including the community hospital LOS) compared to those without AD. Hospital readmission rates were lower for persons with AD. This study also found a shorter LOS (less than 4 days) in an acute care hospital after hip fracture for both cohorts was associated with an increased risk of readmission within 30-days of acute care hospital discharge.

AD has been observed to be a risk for hip fractures [3]. Our findings in regard to total hospital LOS were similar to those found in the systematic review by Möller et al. 2018. The review, pooling together seven studies, reported longer hospital LOS after hip fracture for those with dementia compared to those without dementia, ranging from 0.3 to 10 days longer. The definition of hospital LOS given in the studies varied or no definition was given at all making the results difficult to compare to our study [7]. In Finland, most hip fracture patients are referred to rehabilitation in community hospitals soon after operative treatment in an acute care hospital [22] and the structure of the healthcare system may account for the differences in LOS in general.

Our findings are similar to the mean acute care hospital LOS of 4–5 days for hip fracture in Finland [23]. Treatment and outcomes for hip fracture patients in Finland have improved in recent years, with a decrease in mortality rates and shortened hospital stays [18]. Finland established a National Guideline for care after hip fracture in 2006, [24] possibly leading to care improvements and better outcomes. However, vulnerable patient populations, like older persons with AD, may not be benefiting from the trend of shorter hospital LOS as seen from our results. Shorter LOS may increase the risk of hospital readmissions, as patients are discharged “sicker and quicker” [13]. Shorter acute care hospital stays for patients reduces their exposure to fewer care providers specialized in early postoperative care and for comprehensive evaluations of medical conditions during the initial hospitalization. Comprehensive geriatric assessments, especially when it includes orthogeriatrics, have been found to decrease the risk of complications after hip fracture in older patients [25–27].

In our study those in the AD cohort had fewer hospital readmissions within 30- and 90-days compared to those without AD. A systematic review by Ali and Gibbons 2017 previously found dementia either increased the readmission risk or had no effect [12]. The longer LOS in a community hospital for those with AD may have affected the readmission rates since complications, like post-operative infections, can be treated in community hospitals. Lower readmission rates may also be explained by the higher mortality rates within 90-days of the AD cohort. Dementia has been shown to be an independent predictor of mortality after hip fracture surgery [28]. Our study found higher mortality rates in the AD cohort for within 30- and 90-days of acute care discharge compared to the non-AD cohort. In a large cohort study of older adults in the Sweden, around 5% of persons died within 30 days of discharge after hip fracture [29], which was higher than our findings.

The LOS in an acute care hospital was more strongly associated with readmission within 30-days and 90-days for those with AD than to community hospital LOS and total LOS. A systematic review by Ali and Gibbons 2017 found the effect of hospital LOS for hip fracture on readmission rates have been unclear and few have focused on persons with dementia or AD [12]. The 30-day readmission rate of the AD cohort (10.7%) was similar to the median 30-day readmission rate (10.1%) reported in the review.

The AD cohort had larger proportion of persons in need of continuous or nearly continuous level of assistance at discharge. Rehabilitation outcomes following hip fracture in persons with dementia are dependent on the person’s stage of the disease. Persons with mild to moderate stages of dementia have been found to have similar gains in rehabilitation after hip fracture compared to non-cognitively impaired persons. However, these gains are not seen in the more advanced stages of dementia [30].

In both cohorts, only about 12% were discharged home from the acute care hospital. Other studies have reported between 13 and 34% of older adults discharged home from the hospital after hip fracture [31–33]. In Finnish practice persons and especially older persons who are not able to manage in their homes or who are needing rehabilitation are discharged to community hospitals. The rehabilitation process continues just a few days or in severe cases weeks or even months. This is likely a reflection of differences in care.

Strengths of our study include a nationwide cohort of persons with clinically verified AD diagnosis and accurate hospitalization events and recorded hip fractures [34]. Studies assessing the internal validity of Finnish Care Register for Health Care and comparing register information with patient records or other information from the primary source have confirmed that the coverage and accuracy of these registers are well-suited for epidemiological research [35, 36]. We reported the LOS in acute care and community hospitals separately, which allows the comparison of our results to other countries with different health care systems.

Our study focused on those who were community dwelling at incident of AD diagnosis. Therefore, the results are not entirely generalizable to institutionalized persons. The main limitation of all register-based studies, not specific to ours, is the lack of information on certain confounders, such as lifestyle factors [37]. These lifestyle-based confounders can be partially captured by comorbidities. The AD cohort may not only capture purely AD cases since this can only be done post-mortem autopsy. Some persons in the AD cohort may have mixed dementia. However, the validity of AD diagnosis in the Special Reimbursement register has shown to be fairly accurate, with a positive predictive value of 97.1% [36].

## Conclusion

Shorter LOS in an acute care hospital for hip fracture was associated with an increased risk of hospital readmission within 30-days for both those with and without AD in Finland. Vulnerable populations, such as those with AD, may benefit from a longer acute care hospital LOS after hip fracture possibly giving them access to comprehensive geriatric assessments or orthogeriatric collaboration, and thereby reducing poor health outcomes and costly hospital readmissions.

## Supplementary information

**Additional file 1:.** Figure S1 Determination of length of stay in a community hospital setting and Tables S1-S4: Characteristics of the 30-day and 90-day readmission in AD and non-AD cohorts.

## Data Availability

The data that support the findings of this study are available from the Social Insurance Institution (SII) but restrictions apply to the availability of these data, which were used under license for the current study, and so are not publicly available. Data are however available from the authors upon reasonable request and with permission of SII.

## Abbreviations

AD: Alzheimer’s disease
COPD: Chronic obstructive pulmonary disease
CT: Computed tomography
CVD: Cardiovascular disease
FSRR: Finnish Special Reimbursement Register
ICD: International Classification of Diseases
LOS: Length of stay
MEDALZ study: Medication use and Alzheimer’s disease study
MRI: Magnetic resonance imaging scan
SII: Social Insurance Institution of Finland
