# Supplementary material for: Length of hospital stay after hip fracture and readmission rates of persons with and without Alzheimer’s disease: a matched cohort study
Source: BMC Geriatr. 2020 Jun 18;20:214. doi: 10.1186/s12877-020-01609-5 (PMC7301501; doi:10.1186/s12877-020-01609-5)
Supplement: Supplementary file 1 — Additional file 1:. Figure S1 Determination of length of stay in a community hospital setting and Tables S1-S4: Characteristics of the 30-day and 90-day readmission in AD and non-AD cohorts. [file 12877_2020_1609_MOESM1_ESM.docx]

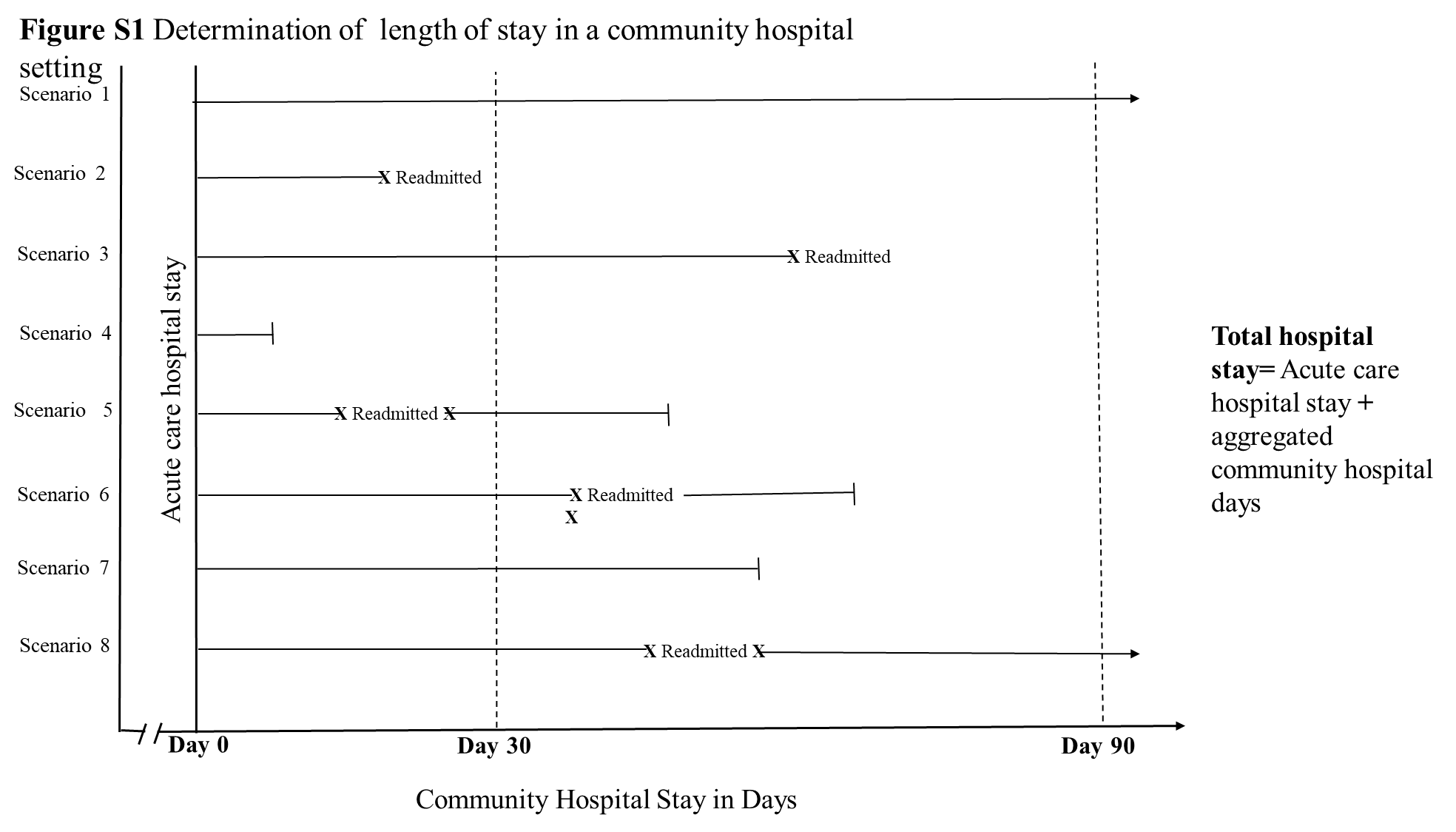


| Table S1 | | | | |
| --- | --- | --- | --- | --- |
| **Characteristics of 30-day readmission in AD cohort** | | | | |
| **Characteristic** | | **30-day Readmission**  (n=672) | **No 30-day Readmission**  (n=5594) | ***P*-Value** |
| Age at hip fracture, mean ± SD | | 84.4 ± 5.4 | 84.6 ± 5.7 | 0.21 |
| Sex | |  |  | <0.001 |
|  | Female, n (%) | 458 (68.2%) | 4357 (77.9%) | ·· |
|  | Male, n (%) | 214 (31.8%) | 1237 (22.1%) | ·· |
| University Hospital District | |  |  | <0.001 |
|  | Helsinki, n (%) | 149 (22.2%) | 1668 (29.8%) | ·· |
|  | Kuopio, n (%) | 141 (21.0%) | 1089 (19.5%) | ·· |
|  | Oulu, n (%) | 121 (18.0%) | 761 (13.6%) | ·· |
|  | Tampere, n (%) | 160 (23.8%) | 1194 (21.3%) | ·· |
|  | Turku, n (%) | 101 (15.0%) | 882 (15.8%) | ·· |
| Year of hip fracture | |  |  | 0.34 |
|  | 2005, n (%) | 9 (1.3%) | 58 (1.0%) | ·· |
|  | 2006, n (%) | 32 (4.8%) | 210 (3.8%) | ·· |
|  | 2007, n (%) | 41 (6.1%) | 316 (5.6%) | ·· |
|  | 2008, n (%) | 73 (10.9%) | 489 (8.7%) | ·· |
|  | 2009, n (%) | 72 (10.7%) | 531 (9.5%) | ·· |
|  | 2010, n (%) | 80 (11.9%) | 689 (12.3%) | ·· |
|  | 2011, n (%) | 92 (13.7%) | 803 (14.4%) | ·· |
|  | 2012, n (%) | 82 (12.2%) | 822 (14.7%) | ·· |
|  | 2013, n (%) | 87 (12.9%) | 701 (12.5%) | ·· |
|  | 2014, n (%) | 53 (7.9%) | 539 (9.6%) | ·· |
|  | 2015, n (%) | 51 (7.6%) | 436 (7.8%) | ·· |
| Median acute care hospital stay, days (IQR) | | 4 (2-6) | 4 (3-7) | <0.001 |
| Discharged to community hospital, n (%) | | 606 (90.2%) | 4880 (89·0%) | 0.030 |
| Median community hospital stay, days (IQR) * | | 40.5 (15-97) | 35(16-83) | 0.18 |
| Median total hospital stay, days (IQR) | | 37.5 (15-93) | 34 (15-80) | 0.71 |
| Diabetes, n (%) | | 96 (14.3%) | 641 (11.5%) | 0.036 |
| Stroke, n (%) | | 72 (10.7%) | 474 (8.5%) | 0.06 |
| CVD, n (%) | | 398 (59.2%) | 2620 (46.8%) | <0.001 |
| Mental disorders, n (%) | | 162 (24.1%) | 1302 (23.3%) | 0.63 |
| Asthma/COPD, n (%) | | 71 (10.6%) | 468 (8.4%) | 0.06 |
| Epilepsy, n (%) | | 26 (3.9%) | 139 (2.9%) | 0.041 |
| Highest occupational social class | |  |  | 0.10 |
|  | Managerial/ Professional, n (%) | 115 (17.1%) | 1055 (18.9%) | ·· |
|  | Office worker, n (%) | 58 (8.6%) | 550 (9.8%) | ·· |
|  | Farming/ forestry, n (%) | 145 (21.6%) | 968 (17.3%) | ·· |
|  | Sales/industry/ cleaning, n (%) | 283 (42.1%) | 2365 (42.3%) | ·· |
|  | Unknown, n (%) | 67 (10.0%) | 604 (10.8%) | ·· |
|  | Did not respond, n (%) | 4 (0.6%) | 52 (0.9%) | ·· |
| Required level of assistance at discharge | |  |  | 0.025 |
|  | Nearly independent, n (%) | 8 (1.2%) | 135 (2.4%) | ·· |
|  | Intermittent need, n (%) | 94 (14.0%) | 708 (12.7%) | ·· |
|  | Recurrent need, n (%) | 276 (41.1%) | 2432 (43.5%) | ·· |
|  | Nearly continuous, n (%) | 164 (24.4%) | 1223 (21.9%) | ·· |
|  | Continuous, n (%) | 111 (16.5%) | 835 (14.9%) | ·· |
|  | Missing data, n (%) | 19 (2.8%) | 261 (4.7%) | ·· |
| Median duration of AD diagnosis, years (IQR) | | 2.7(1.2-4.2) | 2.8 (1.3-4.5) | 0.008 |
| Died within 90-days, n (%) | | 169 (25.1%) | 536 (9.6%) | <0.001 |
| Abbreviations: AD= Alzheimer’s disease; SD= Standard Deviation; IQR= Interquartile Range; CVD= Cardiovascular Disease; COPD= Chronic Obstructive Pulmonary Disease | | | | |
| * only those discharged to a community hospital | | | | |

| Table S2 | | | | |
| --- | --- | --- | --- | --- |
| **Characteristics of 30-day readmission in Non-AD cohort** | | | | |
| **Characteristic** | | **30-day Readmission**  (n=836) | **No 30-day Readmission**  (n=5430) | ***P-*Value** |
| Age at hip fracture, mean ± SD | | 84.3 ± 5.6 | 84.6 ± 5.7 | 0.14 |
| Sex | |  |  |  |
|  | Female, n (%) | 574 (68.7%) | 4241 (78.1%) | <0.001 |
|  | Male, n (%) | 262 (31.3%) | 1189 (21.9%) |  |
| University Hospital District | |  |  | 0.001 |
|  | Helsinki, n (%) | 195 (23.3%) | 1596 (29.4%) | ·· |
|  | Kuopio, n (%) | 180 (21.5%) | 1015 (18.7%) | ·· |
|  | Oulu, n (%) | 137 (16.4%) | 702 (12.9%) | ·· |
|  | Tampere, n (%) | 188 (22.5%) | 1193 (22.0%) | ·· |
|  | Turku, n (%) | 136 (16.3%) | 924 (17.0%) | ·· |
| Year of hip fracture | |  |  | 0.57 |
|  | 2005, n (%) | 11 (1.3%) | 62 (1.1%) | ·· |
|  | 2006, n (%) | 20 (2.4%) | 215 (4.0%) | ·· |
|  | 2007, n (%) | 38 (4.5%) | 320 (5.9%) | ·· |
|  | 2008, n (%) | 77 (9.2%) | 486 (9.0%) | ·· |
|  | 2009, n (%) | 78 (9.3%) | 528 (9.7%) | ·· |
|  | 2010, n (%) | 109 (13.0%) | 678 (12.5%) | ·· |
|  | 2011, n (%) | 121 (14.5%) | 748 (13.8%) | ·· |
|  | 2012, n (%) | 127 (15.2%) | 767 (14.1%) | ·· |
|  | 2013, n (%) | 105 (12.6%) | 678 (12.5%) | ·· |
|  | 2014, n (%) | 81 (9.7%) | 514 (9.5%) | ·· |
|  | 2015, n (%) | 69 (8.3%) | 434 (8.0%) | ·· |
| Median acute care hospital stay, days (IQR) | | 4 (3-6) | 5 (3-7) | <0.001 |
| Discharged to community hospital, n (%) | | 753 (90.1%) | 4730 (86.3%) | 0.016 |
| Median community hospital stay, days (IQR) * | | 32 (14-73) | 29 (15-63) | 0.36 |
| Median total hospital stay, days (IQR) | | 31 (14-72.5) | 29 (15-61) | 0.19 |
|  | |  |  |  |
| Diabetes, n (%) | | 127 (15.2%) | 647 (11.9%) | 0.009 |
| Stroke, n (%) | | 87 (10.4%) | 559 (10.3%) | 0.90 |
| CVD, n (%) | | 475 (56.8%) | 2677 (49.3%) | <0.001 |
| Mental disorders, n (%) | | 120 (14.4%) | 681 (12.5%) | 0.14 |
| Asthma/COPD, n (%) | | 95 (11.4%) | 478 (8.8%) | 0.020 |
| Epilepsy, n (%) | | 27 (3.2%) | 108 (2.0%) | 0.029 |
| Highest occupational social class | |  |  | 0.048 |
|  | Managerial/ Professional, n (%) | 150 (17.9%) | 985 (18.1%) | ·· |
|  | Office worker, n (%) | 72 (8.6%) | 523 (9.6%) | ·· |
|  | Farming/ Forestry, n (%) | 179 (21.4%) | 1079 (19.9%) | ·· |
|  | Sales/ Industry/ Cleaning, n (%) | 350 (41.9%) | 2153 (39.7%) | ·· |
|  | Unknown, n (%) | 83 (9.9%) | 618 (11.4%) | ·· |
|  | Did not respond, n (%) | 2 (0.2%) | 72 (1.3%) | ·· |
| Required level of assistance at discharge | |  |  | <0.001 |
|  | Nearly independent, n (%) | 34 (4.1%) | 291 (5.4%) | ·· |
|  | Intermittent need, n (%) | 153 (18.3%) | 989 (18.2%) | ·· |
|  | Recurrent need, n (%) | 327 (39.1%) | 2468 (45.5%) | ·· |
|  | Nearly continuous, n (%) | 175 (20.9%) | 858 (15.8%) | ·· |
|  | Continuous, n (%) | 116 (13.9%) | 592 (10.9%) | ·· |
|  | Missing data, n (%) | 31 (3.7%) | 232 (4.3%) | ·· |
| Died within 90-days, n (%) | | 177 (21.2%) | 301 (5.5%) | <0.001 |
| Abbreviations: AD= Alzheimer’s disease; SD= Standard Deviation; IQR= Interquartile Range; CVD= Cardiovascular Disease; COPD= Chronic Obstructive Pulmonary Disease | | | | |
| * only those discharged to a community hospital | | | | |
| Table S3 | | | | |
| **Characteristics of 90-day readmission in AD cohort** | | | | |
| **Characteristic** | | **90-day Readmission**  (n=1059) | **No 90-day Readmission**  (n=5207) | ***P*-Value** |
| Age at hip fracture, mean ± SD | | 84.3 ± 5·5 | 84.7 ± 5.7 | 0.056 |
| Sex | |  |  | <0.001 |
|  | Women, n (%) | 742 (70.1%) | 4073 (78.2%) | ·· |
|  | Male, n (%) | 317 (29.9%) | 1134 (22%) | ·· |
| University Hospital District | |  |  | <0.001 |
|  | Helsinki, n (%) | 261 (24.6%) | 1556 (29.9%) | ·· |
|  | Kuopio, n (%) | 227 (21.4%) | 1003 (19.3%) | ·· |
|  | Oulu, n (%) | 184 (17.4%) | 698 (13.4%) | ·· |
|  | Tampere, n (%) | 230 (21.7%) | 1124 (21.6%) | ·· |
|  | Turku, n (%) | 157 (14.8%) | 826 (15.9%) | ·· |
| Year of hip fracture | |  |  | 0.16 |
|  | 2005, n (%) | 13 (1.2%) | 54 (1.0%) | ·· |
|  | 2006, n (%) | 44 (4.2%) | 198 (3.8%) | ·· |
|  | 2007, n (%) | 70 (6.6%) | 287 (5.5%) | ·· |
|  | 2008, n (%) | 111 (10.5%) | 451 (8.7%) | ·· |
|  | 2009, n (%) | 116 (11.0%) | 487 (9.4%) | ·· |
|  | 2010, n (%) | 128 (12.1%) | 641 (12.3%) | ·· |
|  | 2011, n (%) | 146 (13.8%) | 749 (14.4%) | ·· |
|  | 2012, n (%) | 129 (12.2%) | 775 (14.9%) | ·· |
|  | 2013, n (%) | 129 (12.2%) | 659 (12.7%) | ·· |
|  | 2014, n (%) | 90 (8.5%) | 502 (9.6%) | ·· |
|  | 2015, n (%) | 83 (7.8%) | 404 (7.8%) | ·· |
| Median acute care hospital stay, days (IQR) | | 4 (3-7) | 4 (3-7) | 0.004 |
| Discharged to community hospital, n (%) | | 958 (90.5%) | 4528 (87.0%) | 0.002 |
| Median community hospital stay, days (IQR) * | | 39 (17-89) | 35 (15-83) | 0.048 |
| Median total hospital stay, days (IQR) | | 40 (16-89) | 33 (15-80) | 0.003 |
| Diabetes, n (%) | | 154 (14.5%) | 583 (11.2%) | 0.003 |
| Stroke, n (%) | | 112 (10.6%) | 434 (8.3%) | 0.023 |
| CVD, n (%) | | 574 (54.2%) | 2444 (46.9%) | <0.001 |
| Mental disorders, n (%) | | 271 (25.6%) | 1193 (22.9%) | 0.06 |
| Asthma/COPD, n (%) | | 107 (10.1%) | 432 (8.3%) | 0.06 |
| Epilepsy, n (%) | | 33 (3.1%) | 132 (2.5%) | 0.29 |
| Highest occupational social class | |  |  | 0.005 |
|  | Managerial/ Professional, n (%) | 178 (16.8%) | 992 (19.1%) | ·· |
|  | Office worker, n (%) | 88 (8.3%) | 520 (10.0%) | ·· |
|  | Farming/ Forestry, n (%) | 230 (21.7%) | 883 (17.0%) | ·· |
|  | Sales/ Industry/ Cleaning, n (%) | 444 (41.9%) | 2204 (42.3%) | ·· |
|  | Unknown, n (%) | 108 (10.2%) | 563 (10.8%) | ·· |
|  | Did not respond, n (%) | 11 (1.0%) | 45 (0.9%) | ·· |
| Median duration of AD diagnosis, years (IQR) | | 2.7 (1.2-4.2) | 2.8 (1.4-4.5) | 0.011 |
| Required level of assistance at discharge | |  |  | 0.018 |
|  | Nearly independent, n (%) | 15 (1.4%) | 128 (2.5%) | ·· |
|  | Intermittent need, n (%) | 151 (14.3%) | 651 (12.5%) | ·· |
|  | Recurrent need, n (%) | 436 (41.2%) | 2272 (43.6%) | ·· |
|  | Nearly continuous, n (%) | 260 (24.6%) | 1127 (21.6%) | ·· |
|  | Continuous, n (%) | 160 (15.1%) | 786 (15.1%) | ·· |
|  | Missing data, n (%) | 37 (3.5%) | 243 (4.7%) | ·· |
| Abbreviations: AD= Alzheimer’s disease; SD= Standard Deviation; IQR= Interquartile Range; CVD= Cardiovascular Disease; COPD,= Chronic Obstructive Pulmonary Disease | | | | |
| * only those discharged to a community hospital | | | | |

| Table S4 | | | | |
| --- | --- | --- | --- | --- |
| **Characteristics of 90-day readmission in non-AD cohort** | | | | |
| **Characteristic** | | **90-day Readmission**  (n=1300) | **No 90-day Readmission**  (n=4966) | ***P* Value** |
| Age at hip fracture, mean ± SD | | 84.3 ± 5.7 | 84.7 ± 5·7 | 0.057 |
| Sex | |  |  | <0.001 |
|  | Female, n (%) | 940 (72.3%) | 3875 (78.0%) | ·· |
|  | Male, n (%) | 360 (27.7%) | 1091 (22.0%) | ·· |
| University Hospital District | |  |  | 0.002 |
|  | Helsinki, n (%) | 330 (25.4%) | 1461 (29.4%) | ·· |
|  | Kuopio, n (%) | 280 (21.5%) | 915 (18.4%) | ·· |
|  | Oulu, n (%) | 201 (15.5%) | 638 (12.8%) | ·· |
|  | Tampere, n (%) | 276 (21.2%) | 1105 (22.3%) | ·· |
|  | Turku, n (%) | 213 (16.4%) | 847 (17.1%) | ·· |
| Year of hip fracture | |  |  | 0.51 |
|  | 2005, n (%) | 18 (1.4%) | 55 (1.1%) | ·· |
|  | 2006, n (%) | 40 (3.1%) | 195 (3.9%) | ·· |
|  | 2007, n (%) | 64 (4.9%) | 294 (5.9%) | ·· |
|  | 2008, n (%) | 115 (8.8%) | 448 (9.0%) | ·· |
|  | 2009, n (%) | 143 (11.0%) | 463 (9.3%) | ·· |
|  | 2010, n (%) | 172 (13.2%) | 615 (12.4%) | ·· |
|  | 2011, n (%) | 177 (13.6%) | 692 (13.9%) | ·· |
|  | 2012, n (%) | 189 (14.5%) | 705 (14.2%) | ·· |
|  | 2013, n (%) | 167 (12.8%) | 616 (12.4%) | ·· |
|  | 2014, n (%) | 117 (9.0%) | 478 (9.6%) | ·· |
|  | 2015, n (%) | 98 (7.5%) | 405 (8.2%) | ·· |
| Median acute care hospital stay, days (IQR) | | 4 (3-7) | 5 (3-7) | 0.007 |
| Discharged to community hospital, n (%) | | 1179 (90·7%) | 4304 (86·7%) | <0.001 |
| Median community hospital stay, days (IQR) | | 33 (33-70·5) | 28 (14-63) | 0.027 |
| Median total hospital stay, days (IQR) * | | 34 (16-70) | 29 (15-60) | <0.001 |
| Diabetes, n (%) | | 195 (15.0%) | 579 (11.7%) | 0.001 |
| Stroke, n (%) | | 138 (10.6%) | 508 (10.2%) | 0.68 |
| CVD, n (%) | | 722 (55.5%) | 2430 (48.9%) | <0.001 |
| Mental disorders, n (%) | | 175 (13.5%) | 626 (12.6%) | 0.42 |
| Asthma/COPD, n (%) | | 134 (10.3%) | 439 (8.8%) | 0.11 |
| Epilepsy, n (%) | | 41 (3.2%) | 94 (1.9%) | 0.007 |
| Highest occupational Social class | |  |  | 0.013 |
|  | Managerial/ Professional, n (%) | 224 (17.2%) | 911 (18.3%) | ·· |
|  | Office worker, n (%) | 109 (8.4%) | 486 (9.8%) | ·· |
|  | Farming/ Forestry, n (%) | 281 (21.6%) | 977 (19.7%) | ·· |
|  | Sales/ Industry/ Cleaning, n (%) | 545 (41.9%) | 1958 (39.4%) | ·· |
|  | Unknown, n (%) | 135 (10.4%) | 566 (11.4%) | ·· |
|  | Did not respond, n (%) | 6 (0.5%) | 68 (1.4%) | ·· |
| Required level of assistance at discharge | |  |  | 0.004 |
|  | Nearly independent, n (%) | 51 (3.9%) | 274 (5.5%) | ·· |
|  | Intermittent need, n (%) | 256 (19.7%) | 886 (17.8%) | ·· |
|  | Recurrent need, n (%) | 545 (41.9%) | 2250 (45.3%) | ·· |
|  | Nearly continuous, n (%) | 246 (18.9%) | 787 (15.8%) | ·· |
|  | Continuous, n (%) | 155 (11.9%) | 553 (11.1%) | ·· |
|  | Missing data, n (%) | 47 (3.6%) | 216 (4.3%) | ·· |
| Abbreviations: AD= Alzheimer’s disease; SD= Standard Deviation; IQR= Interquartile Range; CVD= Cardiovascular Disease; COPD= Chronic Obstructive Pulmonary Disease | | | | |
| * only those discharged to a community hospital | | | | |
